# Dissociating verbal and nonverbal audiovisual object processing

**DOI:** 10.1016/j.bandl.2008.10.005

**Published:** 2009-02

**Authors:** Julia Hocking, Cathy J. Price

**Affiliations:** a Wellcome Trust Centre for Neuroimaging, UCL, London, UK; b The University of Queensland, Centre for Magnetic Resonance, Brisbane, Australia

**Keywords:** Audiovisual, Integration, Verbal, Nonverbal, Semantic, Conceptual, Phonological, Amodal

## Abstract

This fMRI study investigates how audiovisual integration differs for verbal stimuli that can be matched at a phonological level and nonverbal stimuli that can be matched at a semantic level. Subjects were presented simultaneously with one visual and one auditory stimulus and were instructed to decide whether these stimuli referred to the same object or not. Verbal stimuli were simultaneously presented spoken and written object names, and nonverbal stimuli were photographs of objects simultaneously presented with naturally occurring object sounds. Stimulus differences were controlled by including two further conditions that paired photographs of objects with spoken words and object sounds with written words. Verbal matching, relative to all other conditions, increased activation in a region of the left superior temporal sulcus that has previously been associated with phonological processing. Nonverbal matching, relative to all other conditions, increased activation in a right fusiform region that has previously been associated with structural and conceptual object processing. Thus, we demonstrate how brain activation for audiovisual integration depends on the verbal content of the stimuli, even when stimulus and task processing differences are controlled.

## 1 Introduction

Previous functional imaging studies investigating audiovisual object processing have used either verbal (Bernstein, Auer, Wagner, & Ponton, 2008; Calvert, Campbell, & Brammer, 2000; Kreifelts, Ethofer, Grodd, Erb, & Wildgruber, 2007; Macaluso, George, Dolan, Spence, & Driver, 2004; Raij, Uutela, & Hari, 2000; Sekiyama, Kanno, Miura, & Sugita, 2003; Skipper, Nusbaum, & Small, 2005; Skipper, van Wassenhove, Nusbaum, & Small, 2007; van Atteveldt, Formisano, Blomert, & Goebel, 2007; van Atteveldt, Formisano, Goebel, & Blomert, 2004; Wright, Pelphrey, Allison, McKeown, & McCarthy, 2003) or nonverbal (Beauchamp, Lee, Argall, & Martin, 2004; Sestieri et al., 2006; Taylor, Moss, Stamatakis, & Tyler, 2006) stimuli. By verbal versus nonverbal, we refer to the presence or absence of word stimuli—whether written, spoken or lip-read. The focus of the present paper is on how neuronal activation for audiovisual processing differs for verbal versus nonverbal conceptual stimuli.

Our predictions are based on the following rationale. Verbal and nonverbal conceptual stimuli can access both phonological and semantic processes; however they do so in different ways. For verbal stimuli, phonetic analysis of the input is required before recognition at the semantic level (e.g. Indefrey and Levelt, 2004). By contrast, for nonverbal stimuli, semantic processing is required before phonological retrieval (e.g. Glaser & Glaser, 1989; Seifert, 1997). Consequently, audiovisual matching of two verbal stimuli (i.e. auditory and visual words) can occur at the level of phonology prior to explicit retrieval of semantics whereas audiovisual matching of two nonverbal stimuli (e.g. pictures and sounds of objects) can occur at the level of semantics without explicit retrieval of phonology. This leads us to predict that activation in phonological processing areas may be higher for matching verbal than nonverbal stimuli whereas activation in semantic processing areas may be higher for matching nonverbal than verbal stimuli. In addition, reports of brain damaged patients with selective deficits in either verbal or nonverbal stimuli have suggested that there may be verbal and nonverbal dissociations within the semantic system. Depending on the theoretical perspective taken, this dissociation has been proposed at the level of (i) separate visual and verbal semantic systems (e.g. Ferreira, Giusiano, Ceccaldi, & Poncet, 1997; Warrington, 1975; Warrington & McCarthy, 1994), (ii) a shared distributed semantic system differentiated by the type of knowledge primarily involved during acquisition (e.g. Saffran, Coslett, & Keener, 2003) or (iii) differences at the level that verbal and nonverbal stimuli access a shared semantic system.

Verbal (word) stimuli can either be presented in the form of continuous speech (as in Calvert et al., 2000; Macaluso et al., 2004) or in the form of single words (Ojanen et al., 2005; Olson, Gatenby, & Gore, 2002; Raij et al., 2000; van Atteveldt et al., 2004; van Atteveldt et al., 2007). The verbal stimuli used in this experiment were written and spoken object names because this permitted a controlled comparison to nonverbal audiovisual stimuli in the form of pictures of objects and the naturally occurring sounds associated with objects. The task was held constant and involved deciding if a visually presented stimulus referred to the same object as a simultaneously presented auditory stimulus. If verbal versus nonverbal audiovisual object matching differ in the relative demands they place on phonological and semantic processing (see above) then we would expect to see fMRI activation differences in areas that have previously been associated with phonological and semantic processing. In contrast if verbal and nonverbal audiovisual object matching depend on different types of semantic processing then we need to consider the results of previous studies that compared verbal and nonverbal semantic/conceptual processing. Below, we briefly review the relevant literature on verbal and nonverbal processing and the influence of these findings on our anatomical expectations.

### 1.1 Phonological versus semantic processing

Functional imaging studies comparing phonological to semantic processing have associated phonological processing with the left superior temporal sulcus (Binder, 2000; Noppeney, Josephs, Hocking, Price, & Friston, 2008; Scott, Blank, Rosen, & Wise, 2000; Wise et al., 2001), the left supramarginal gyrus and left posterior inferior frontal regions (Booth et al., 2006; Demonet et al., 1992; Devlin, Matthews, & Rushworth, 2003; Gold, Balota, Kirchhoff, & Buckner, 2005; Mummery, Shallice, & Price, 1999; Paulesu, Frith, & Frackowiak, 1993; Price, Mummery, Moore, Frakowiak, & Friston, 1999; Roskies, Fiez, Balota, Raichle, & Petersen, 2001). We therefore predicted that activation in one or more of these regions would be higher for audiovisual matching of verbal relative to nonverbal conceptual stimuli. In contrast, semantic processing has been associated with the left middle temporal gyrus (Binder et al., 1997), left anterior temporal lobe (Scott et al., 2000; Vandenberghe, Price, Wise, Josephs, & Frackowiak, 1996), the angular gyri (Devlin et al., 2003; Mummery et al., 1999; Noppeney et al., 2008) and ventral and anterior frontal regions (Binder et al., 1997; Booth et al., 2006; Devlin et al., 2003; Gold et al., 2005; Poldrack et al., 1999; Roskies et al., 2001). Activation in one or more of these regions was therefore predicted to be higher for audiovisual matching of nonverbal than verbal conceptual stimuli. For a review of phonological and semantic areas, see Vigneau et al. (2006).

### 1.2 Verbal versus nonverbal semantics

Studies of brain damaged patients have suggested that the left hemisphere may be more engaged in accessing verbal information while the right hemisphere may be more engaged in accessing nonverbal information (Coslett & Saffran, 1992; for reviews see Caramazza, Hillis, Rapp, & Romani, 1990 and Lambon-Ralph et al., 1999). Unfortunately, most of the evidence from patient data comes from a comparison of verbal/nonverbal processing in the visual modality only. Therefore, the conclusions concerning amodal or separable verbal/nonverbal systems are limited.

Recently, functional neuroimaging of normal subjects has provided another source of evidence for a dissociation between verbal and nonverbal processing within either the auditory modality or the visual modality (Adams & Janata, 2002; Bright, Moss, & Tyler, 2004; Chee et al., 2000; Dick et al., 2007; Giraud & Price, 2001; Humphries, Willard, Buchsbaum, & Hickok, 2001; Perani et al., 1999; Thierry, Giraud, & Price, 2003; Thierry & Price, 2006; Vandenberghe et al., 1996; von Kriegstein, Eger, Kleinschmidt, & Giraud, 2003). Critically, however, the areas associated with verbal and nonverbal stimuli differ according to the input modality (visual or auditory). These between-modality differences are difficult to interpret because they are confounded by perceptual differences in the nature of the verbal and nonverbal stimuli. To circumvent perceptual confounds, a study by Thierry and Price (2006) looked for verbal versus nonverbal processing differences that were independent of stimulus modality. Combining data from one experiment using auditory stimuli and another using the corresponding visual stimuli, they reported a left/right double dissociation for verbal/nonverbal material, independent of sensory modality. Specifically, verbal relative to nonverbal material activated anterior and posterior regions of the left superior temporal sulcus and the ventral left inferior frontal gyrus, while nonverbal relative to verbal material activated the right mid fusiform gyrus and right posterior middle temporal gyrus.

The anatomical dissociation reported in Thierry and Price (2006) provides hypotheses for the current experiment. However, it should still be noted that the functional level at which the verbal versus nonverbal differences arise in Thierry and Price (2006) is debatable. For example, the right posterior superior temporal region associated with nonverbal conceptual processing in Thierry and Price (2006) has been associated with spatial localisation in both the auditory (Rauschecker, 1998a; Rauschecker, 1998b; Rauschecker & Tian, 2000) and visual (Milner & Goodale, 1993) domains. Conversely, the auditory and visual verbal stimuli used in Thierry and Price (2006) had a sentence like structure which may have evoked morpho-syntactic associations compared to nonverbal stimuli. Indeed, the left anterior superior temporal cortex that was activated for verbal relative to nonverbal conditions in Thierry and Price (2006) has previously been associated with morpho-syntactic processing (Bornkessel, Zysset, Friederici, von Cramon, & Schlesewsky, 2005; Dronkers, 2000; Dronkers and Ogar, 2004; Friederici & Kotz, 2003; Friederici, Ruschemeyer, Hahne, & Fiebach, 2003; Humphries, Love, Swinney, & Hickok, 2005; Stowe et al., 1999; Vandenberghe, Nobre, & Price, 2002).

In summary, the present study contrasts the effects of matching verbal versus nonverbal simultaneously presented audiovisual pairs by manipulating the type of input material. Purely verbal audiovisual stimuli were simultaneously presented spoken and written object names, purely nonverbal stimuli were photographs of objects simultaneously presented with naturally occurring object sounds. Perceptual differences between verbal and nonverbal stimuli were controlled by including audiovisual conditions that presented one verbal and one nonverbal stimulus (spoken names with photographs or written names with object sounds). The predictions were that, verbal stimuli would increase activation in left hemisphere areas associated with phonological processing whereas nonverbal stimuli would increase activation in semantic processing areas (possibly in the right hemisphere).

## 2 Materials and methods

### 2.1 Subjects

Eighteen subjects participated in this Experiment (12 women, 6 men, age range 20–36 years, mean age 26). All were right handed native English speakers with normal or corrected to normal vision and gave informed consent to take part. All had normal neurological and audiological status. The study was approved by the joint ethics committee of the Institute of Neurology and University College London Hospital, London, UK.

### 2.2 Experimental design

Subjects were presented bimodally with two simultaneously presented conceptual stimuli, one in the visual modality (colour photograph or written object name) and one in the auditory modality (spoken object name or object sound). The task was to indicate, via a left or right hand key pad response (counterbalanced across subjects), whether the two stimuli within each trial referred to the same object or not.

There were four possible audiovisual combinations:

1) Verbal stimuli (VV): Auditory object names with written object names.
2) Nonverbal stimuli (NN): Auditory object sounds with photographs of objects.
3) Verbal and Nonverbal (VN): Auditory object names with photographs of objects.
4) Nonverbal and Verbal (NV): Auditory object sounds with written object names.

These four conditions varied in their verbal content: condition 1 presented two verbal stimuli, conditions 3 and 4 presented one verbal stimulus, and condition 2 had no verbal stimuli. The mixed verbal-nonverbal pairs (VN and NV), therefore served two purposes. First they allowed us to look for a graded effect of verbal content (2 > 1 > 0 verbal stimuli), second they allowed us to control for physical differences in stimulus modality. Photographs and object sounds, for example, have different perceptual demands than written and auditory words. These stimulus confounds are removed by looking for areas where activation was either higher for VV than all other conditions or higher for NN than all other conditions.

The nature of the task (equating two stimuli at the conceptual level) generated an additional variable whereby stimuli within each audiovisual pair could either be congruent (refer to the same object) or incongruent (refer to two different objects). Thus there were a total of 8 conditions (the four listed above × congruent and incongruent) which were treated as a two way ANOVA. The first factor was verbal content and the second factor was congruency.

### 2.3 Stimuli

**Fig. 1:**
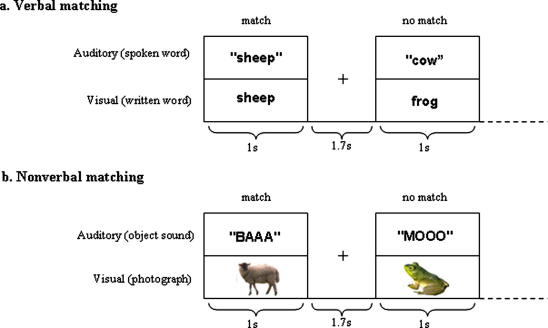
Stimulus trials for simultaneous audiovisual matching, (a) stimulus trials with maximum verbal material, and (b) stimulus trials with no verbal material. Subjects made a “yes they match” or “no they don’t match” response using a keypad depending on whether auditory and visual stimuli referred to the same object or not.

All stimuli referred to the same set of 108 items: 36 animals, 36 objects and 36 musical instruments. Photographs were obtained from the Hemara Photo Objects CD collection; object sounds were downloaded from the internet, with the majority obtained from the website www.sounddogs.com. Spoken words were recorded by a female English speaker in a sound proof room, and written words were presented in Arial font, subtending a viewing angle of 2.0–7.0° (width) × 1.7° (maximum height). Visual stimuli were presented using a rear projector viewed via a mirror mounted on the head coil. All sounds were presented in mono via MRI-compatible electrostatic headphones and normalised using a low-pass 4th order Butterworth filter at 5000 Hz. All stimuli were 1000 ms in duration, except for spoken words which had a range of 650–1000 ms. See Fig. 1 for schematic overview of the experiments.

Stimulus conditions were blocked with 3 congruent and 3 incongruent trials per block. Congruent and incongruent trials were randomized within block. Trial length was 2.7 (1 s stimulus duration followed by 1.7 s fixation), block length was 16.2 s (2.7 × 3 congruent and 3 incongruent trials), fixation after each block alternated between 1.62 and 16.2 s and there were a total of 24 blocks in each of four different scanning sessions. The effects of interest were (1) the number of verbal components in crossmodal integration, and (2) the influence of congruency on these pairings.

### 2.4 Data acquisition

All Data were acquired on a Siemens 1.5 T scanner (Siemens, Erlangen, Germany). Functional images used a T2*-weighted echo-planar (EPI) sequence for BOLD contrast with 3 × 3 mm in plane resolution, 2 mm slice thickness and a 1 mm slice interval. 36 slices were collected, resulting in an effective repetition time (TR) of 3.24 s/volume. After the functional sessions, a T1-weighted anatomical volume image was acquired from all subjects to ensure normal neurological status.

### 2.5 Data analysis

Functional data were analysed with statistical parametric mapping (SPM2, Wellcome Trust Centre for Neuroimaging, London, UK) implemented in Matlab 7.1 (Mathworks, Sherborne, MA, USA). Pre-processing included realignment and unwarping using the first volume as the reference scan (after excluding the first four dummy scans to allow for T1 equilibration effects), spatial normalisation to a standard MNI template (Friston et al., 1995), and spatial smoothing using a 6mm full width half maximum isotropic Gaussian kernel. One subject was removed from the analysis due to excess head movement.

First level statistical analyses (single subject and fixed effects) modelled each trial type independently by convolving the onset times with the haemodynamic response function (Mechelli, Henson, Price, & Friston, 2003). The data were high-pass filtered using a set of discrete cosine basis functions with a cut-off period of 128 s. There were 8 trial types: 4 conditions × 2 congruency. These 8 parameter estimates were then fed into a second level ANOVA with a correction for non-sphericity. The analysis enabled the investigation of:

1. The main effect of all audiovisual pairs relative to fixation.
2. The effect of (i) increasing and (ii) decreasing verbal content.
3. The effect of congruency (congruent versus incongruent).
4. The interaction between congruency and verbal content.

The contrast for increasing verbal content weighted the condition with the number of verbal components (i.e. VV = 2; VN = 1; NV = 1; NN = −4) for both congruent and incongruent conditions. The contrast for decreasing verbal content was the reverse (VV = −2; VN = −1; NV = −1; NN = 4). As described above, differences between verbal and nonverbal conditions are confounded by stimulus differences. To dissociate phonological and semantic effects from stimulus differences, we therefore focus on verbal areas where activation was higher for VV stimuli than all other conditions (NN, VN, NV). This was achieved by identifying where there was a significant effect of increasing (or decreasing) verbal content (at *p* < .05 corrected for multiple comparisons across the whole brain) and then, within these areas, conducting post hoc tests to compare the purely verbal (VV) or purely nonverbal (NN) conditions to all other conditions.

## 3 Results

### 3.1 Behavioural results

Reaction times were analysed using a repeated measures ANOVA, modelling presentation modality and congruency. Means and standard deviation are shown in Table 1. The 4 × 2 ANOVA (4 types of matching, 2 congruency) identified a main effect of condition (*F*[1, 17]) = 32.564, *p* < .0005). Post hoc analysis of this effect revealed faster responses when the stimuli included (1) spoken words than object sounds (*t* = 12.999; *p* < .0005) and (2) written words than pictures (*t* = 5.631; *p* < .0005). As a consequence of this additive effect, response latencies were faster for purely verbal audiovisual pairs (VV in Table 1) relative to all other conditions. There was no main effect of congruency, but there was an interaction between presentation modality and congruency (*F*[3, 51] = 15.675, *p* < .0005). This effect was driven by faster responses to congruent than incongruent pairs, but only for the purely verbal condition (*t* = −5.986; *p* < .0005) with no effect of congruency in any other condition (*p* > .05).

### 3.2 Functional Imaging results

#### 3.2.1 The main effect of all audiovisual pairs relative to fixation

**Fig. 2:**
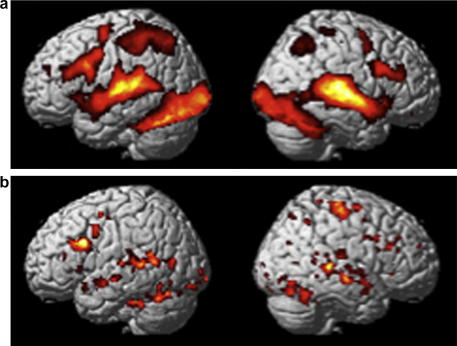
Activation for audiovisual object matching. (a) Common effects for all conditions relative to the fixation baseline, rendered on the SPM standard surface model of an averaged brain at *p* < .05 corrected for multiple comparisons. (b) Increased activation for incongruent relative to congruent pairs within all regions activated for audiovisual matching. Rendered at *p* < .05, uncorrected in areas activated in (a).

All conditions activated a bilateral network of occipital, temporal, parietal and frontal regions (See Fig. 2a). This network reflects all stages of the object matching task from early sensory audiovisual input, through semantic processing, decision-making, motor preparation and response execution.

#### 3.2.2 The effect of increasing verbal information on audiovisual matching

**Fig. 3:**
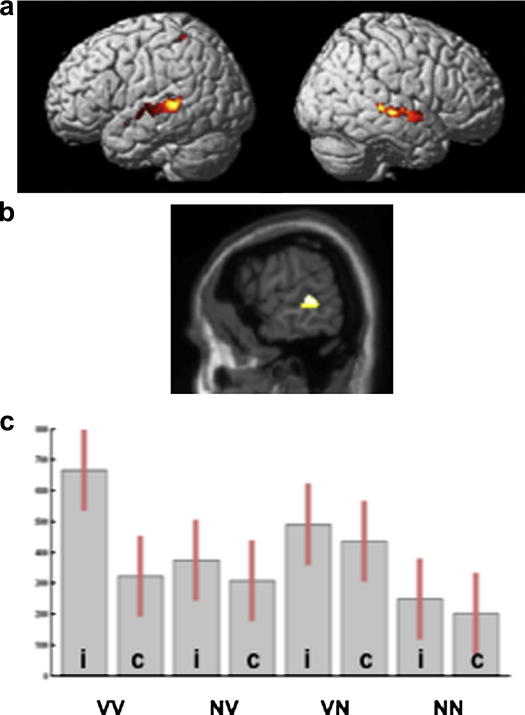
Verbal > nonverbal audiovisual object matching. The effect of increasing verbal content on audiovisual matching (a) rendered at *p* < .001, uncorrected and (b) on a saggital slice (*x* = −62), to illustrate the peak at [−62, −36, 4]. (c) Plot of parameter estimate at [−62, −36, 4] showing highest activation for incongruent verbal stimuli, Key: V, verbal; N, nonverbal; i, incongruent trials; c, congruent trials.

Increasing verbal content increased activation in bilateral superior temporal gyri/sulci (see Fig. 3 and Table 2) with one peak in the left superior temporal sulcus at [−62, −36, 4; *Z* = 4.9] reaching a level of significance that was corrected for multiple comparisons across the whole brain. Post hoc comparison of the different conditions indicated that the effect of increasing verbal content was driven by incongruent verbal stimuli (VV) with higher activation for this condition relative to all other conditions (at *p* < .05 uncorrected) and least activated for congruent and incongruent nonverbal stimuli (NN) than all other conditions, (see Fig. 3).

#### 3.2.3 The effect of decreasing verbal information (i.e. increased for nonverbal)

**Fig. 4:**
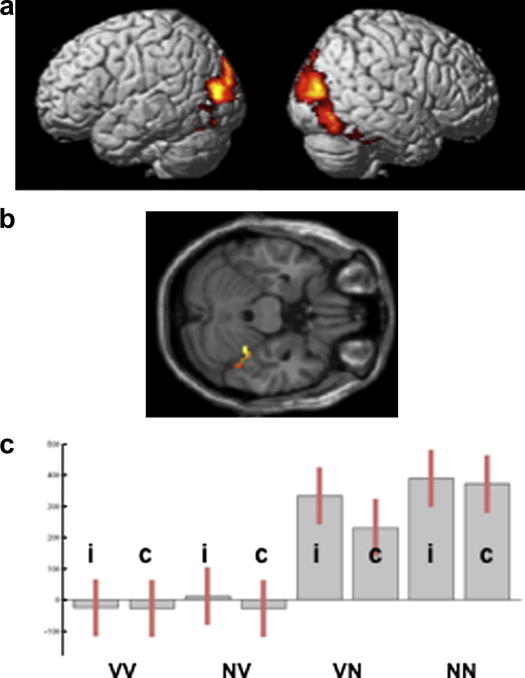
Nonverbal > verbal audiovisual matching. The effect of increasing nonverbal content on audiovisual matching (a) rendered at *p* < .001, uncorrected; and (b) on an axial plane (*z* = −22) to show the medial and lateral sub-peaks at [30, −42, −24], and [−42 −46 −24], respectively. c. Plot of parameter estimate at [30, −42, −24], see Fig. 3 for abbreviations.

Decreasing verbal content increased activation in bilateral occipito-temporal regions (see Table 3 for co-ordinates of significant clusters) but only one cluster in the right middle fusiform [peak at 30, −42, −24; *Z* = 5.7] (see Table 4) was more activated by the purely nonverbal condition (pictures and environmental sounds) relative to each of the other conditions. Within this cluster, there was a second peak at [42, −46, −22; *Z* = 4.3] that corresponded almost exactly to the nonverbal conceptual area reported in Thierry and Price [46, −46, −22]. Both peaks are shown on the axial slice in Fig. 4.

#### 3.2.4 The effect of congruency on audiovisual matching

Nothing reached a corrected level of significance for congruent versus incongruent pairs across the whole brain. However, if we lowered the statistical threshold in areas activated in the main effect of audiovisual matching relative to fixation, then the majority of this system was more activated for incongruent than congruent trials (see Fig. 2b). This included the left posterior superior temporal region that was sensitive to increasing verbal input (−56, −42, 6; *Z* = 3.1; *p* < .001).

#### 3.2.5 The interaction of congruency and verbal input

Nothing reached a corrected level of significance across the whole brain. When the statistical threshold was lowered in areas that activated in the main effect of audiovisual matching relative to fixation, there was a weak trend for the incongruency effect to be greater for verbal than nonverbal pairs in the left posterior superior temporal area (−60, −38, 2; *Z* = 2.3; *p* = .01 uncorrected). This trend is consistent with the behavioural data.

## 4 Discussion

The current study investigated the effect of verbal versus nonverbal material on activation during an audiovisual object matching task. To summarise, within the bilateral network activated during audiovisual matching across all pair types, activation was modulated by the level of verbal content. When verbal material was maximal, activation increased in the left superior temporal sulcus and decreased in the right fusiform gyrus. This was accompanied by a decrease in response times for purely verbal stimuli. There were no areas where activation was higher for congruent than incongruent stimuli but there was a trend for all audiovisual matching regions to be more activated for incongruent than congruent stimuli.

### 4.1 Verbal audiovisual matching

Our *a priori* prediction was that purely verbal stimuli could be matched at a phonological level whereas purely nonverbal stimuli could be matched at a semantic level. Therefore differential activation for verbal and nonverbal stimuli would be observed in phonological and semantic processing regions respectively. Remarkably, the peak co-ordinates for the effect of verbal versus nonverbal audiovisual matching [−62, −36, 4] were identical to those reported by Thierry and Price (2006) for verbal relative to nonverbal semantic processing within both the visual or auditory domain [−62, −36, 4]. The same area has been associated with amodal phonological processing in a number of different studies (Blank, Scott, Murphy, Warburton, & Wise, 2002; Hickok, Buchsbaum, Humphries, & Muftuler, 2003; Okada, Smith, Humphries, & Hickok, 2003; Price et al., 2006; Warren, Wise, & Warren, 2005; Wise et al., 2001). In other words, the results reported here confirm the prediction that phonological processes play a greater role in verbal than nonverbal audiovisual matching.

Response times for audiovisual matching were faster for congruent verbal stimuli than all other conditions (see Table 1) consistent with the hypothesis that audiovisual matching was facilitated by phonological processing. However, left posterior superior temporal activation was not highest when there was a successful (congruent) verbal match. Therefore, activation in this region does not reflect the active integration of audiovisual verbal stimuli. Instead, we show that left posterior superior temporal activation accurately reflects the total verbal content of the stimuli that was highest for two different (incongruent) verbal inputs and lowest when there were no verbal components (i.e. the purely nonverbal conditions).

### 4.2 Nonverbal audiovisual matching

Given the remarkable correspondence of the verbal effects reported here for audiovisual matching with the left superior temporal region reported for verbal versus nonverbal processing in Thierry and Price (2006) for within modality semantic tasks, one might also predict that there would be a correspondence for the nonverbal effects. Indeed, nonverbal relative to verbal audiovisual matching increased activation in a right fusiform cluster with a sub-peak at [42, −46, −22] that was only 4mm from the co-ordinates [46, −46, −22] reported by Thierry and Price (2006) for nonverbal relative to verbal conceptual decisions within modality.

The most significant nonverbal peak, however, lay more medially at [30, −42, −24]. Activation close to these co-ordinates has been reported by several studies to be higher for processing pictures of artifacts than pictures of animals (Chao, Haxby, & Martin, 1999; Chao, Weisberg, & Martin, 2002; Mechelli, Sartori, Orlandi, & Price, 2006; Noppeney, Price, Penny, & Friston, 2006). Noppeney et al. (2006) proposed that these object category effects were primarily driven by pictorial stimuli and mediated by bottom up processing. Nevertheless, there are reports of similar category effects when the stimuli are written names (Chao et al., 1999; Devlin, Rushworth, & Matthews, 2005). This suggests that the effects are not entirely driven by the perceptual input. One interpretation of the medial fusiform category effects is that activation reflects the semantic relevance of an object’s visual features (see Mechelli et al., 2006). Semantic relevance is a measure of the distinctiveness and importance of an object’s features, that is, concepts may have many semantic features but only a small number of features are relevant for distinguishing it from closely related concepts. For example, “trunk” is a feature with high semantic relevance for the concept “elephant”, whereas “has four legs” is of low semantic relevance for defining the concept “elephant” because this feature is used to define many (both living and non-living) concepts.

The current study shows that, although activation at [30, −42, −24] was primarily driven by picture stimuli, activation is higher when pictures are matched to sounds than when pictures are matched to written words (see Fig. 4 and Table 4). One possibility is that the demands on bottom up structural processing are reduced in the presence of spoken words than object sounds. This is consistent with response times being more than 100 ms faster when photographs were presented with auditory names than auditory object sounds (see VN versus NN in Table 1). However, faster response times and reduced structural processing could simply reflect the fact that spoken words were recognised before object sounds which would have the effect of speeding up the matching process and lessening the demands on picture processing.

An alternative perspective is that increased right fusiform activation for nonverbal matching reflects access to stored knowledge of object structure rather than perceptual processing of object structure. This would be consistent with the “semantic processing” conclusions of Chao et al. (1999) following their observation of category effects in these regions during written word processing. Clearly further experiments are required to test the structural versus semantic hypotheses. For example, the structural processing hypothesis could be tested with an experiment that manipulated the timing of the auditory stimuli so that object sounds were recognised at the same time point as spoken words. If activation in this region was still higher for the purely nonverbal stimuli, this would be more consistent with the semantic than perceptual hypothesis. Conversely, if the nonverbal effect was lost, this would be more consistent with the structural processing hypothesis.

### 4.3 The effect of congruency

Previous studies have reported increased activation for congruent relative to incongruent audiovisual stimuli (Calvert et al., 2000; Macaluso et al., 2004; van Atteveldt et al., 2007) but these congruency effects are not consistently observed across all tasks (see Hocking & Price, 2008). In the present study, subjects were forced to attend to both visual and auditory modalities in order to decide whether they referred to the same object or not. We found that, in this context, activation was higher for incongruent than congruent stimuli across the whole network of regions involved in the audiovisual matching task. Our explanation is that incongruent trials effectively carry double the conceptual and phonetic information than congruent trials, i.e. two different object concepts during incongruent trials compared with one concept during congruent trials.

### 4.4 Summary

In conclusion, audiovisual matching was faster when the stimuli were verbal than nonverbal. This behavioural observation was accompanied by a double dissociation in activation for verbal versus nonverbal audiovisual matching. Verbal audiovisual matching increased activation in a left posterior superior temporal region associated with phonological processing while nonverbal audiovisual matching increased activation in a right fusiform area that has previously been associated with nonverbal conceptual and structural object processing.

## Figures and Tables

**Table 1:** Behavioural data.

Audiovisual condition	Verbal input	Mean (msec)	SD
VV inc.	2	834	161
VV con.	2	776	166
NV inc.	1	946	176
NV con.	1	945	191
VN inc.	1	870	146
VN con.	1	875	153
NN inc.	0	950	169
NN con.	0	964	195

Table gives means and standard deviation for reaction times in response to audiovisual matching tasks with different levels of verbal input for congruent and incongruent trials. Key: V, verbal; N, nonverbal; inc., incongruent trials; con., congruent trials. SD, standard deviation; msec, milliseconds.

**Table 2:** Main effect of verbal matching.

Anatomical region		*x*	*y*	*z*	Z-score
Left STS/STG:	**Posterior**	**−62**	**−36**	**4**	**4.9**
	Middle	−64	−22	−2	4.0
	Anterior	−58	−8	−6	3.4

Right STS/STG:	Posterior	64	−2	−6	4.7
	Middle	66	−14	−2	4.6
	Anterior	58	−24	−2	4.2

Table shows anatomical regions, MNI co-ordinates and corresponding Z-scores for significant clusters of activation for the main parametric effect of verbal stimuli. Key: STS, superior temporal sulcus; STG, superior temporal gyrus. Bold font highlights the peak that was significant in height after correction for multiple comparisons across the whole brain.

**Table 3:** Main effect of nonverbal matching.

Anatomical region	*x*	*y*	*z*	Z-score
Right fusiform gyrus	30	−42	−22	6.1
	30	−54	−16	5.7
	32	−62	−14	5.5

Left fusiform gyrus	−28	−60	−14	6.0
	−28	−78	−10	4.8
	−30	−42	−20	3.5

Right middle occipital gyrus	32	−94	10	5.7
	42	−90	4	3.2

Left middle occipital gyrus	−30	−94	18	5.3
	−36	−86	6	4.2
	−44	−84	4	4.0

The anatomical regions, MNI co-ordinates and corresponding Z-scores for activation that increased with decreasing verbal input.

**Table 4:** Nonverbal effects in the right fusiform.

Nonverbal input				NN > NV				NN > VN				NN > VV			
*x*	*y*	*z*		*x*	*y*	*z*		*x*	*y*	*z*		*x*	*y*	*z*	
30	−42	−24	(5.7)	30	−44	−22	(7.0)	32	−42	−24	(1.8)	30	−42	−24	(7.3)
42	−46	−22	(4.3)	42	42	22	(5.3)	42	46	22	(2.0)	42	46	22	(4.5)

Table shows co-ordinates and corresponding Z-scores (in parentheses) for the effect of matching with no verbal information (NN) relative to all other conditions, using a 6 mm search volume based on the two peaks in the right fusiform peak.
